# Cyclic Lipopeptides of *Bacillus amyloliquefaciens* DHA6 Are the Determinants to Suppress Watermelon Fusarium Wilt by Direct Antifungal Activity and Host Defense Modulation

**DOI:** 10.3390/jof9060687

**Published:** 2023-06-19

**Authors:** Dhabyan Mutar Kareem Al-Mutar, Muhammad Noman, Noor Salih Abduljaleel Alzawar, Azizullah, Dayong Li, Fengming Song

**Affiliations:** 1 Key Laboratory of Crop Diseases and Insect Pests of Ministry of Agriculture, Institute of Biotechnology, Zhejiang University, Hangzhou 310058, China; dhabyan@zju.edu.cn (D.M.K.A.-M.); m.noman@zju.edu.cn (M.N.); azizullahkeerio0706@yahoo.com (A.); dyli@zju.edu.cn (D.L.); 2 Key Laboratory of Biology of Crop Pathogens and Insects of Zhejiang Province, Institute of Biotechnology, Zhejiang University, Hangzhou 310058, China; 3 Basra Agriculture Directorate, Almudaina 61008, Iraq; 4 Ministry of Agriculture, Directorate of Agriculture Extension and Training, Albasra 61001, Iraq; masterbiology85@gmail.com

**Keywords:** biocontrol, extracellular lipopeptides, *Fusarium oxysporum* f. sp. *niveum*, Fusarium wilt, induced systemic resistance, watermelon

## Abstract

Fusarium wilt, caused by *Fusarium oxysporum* f. sp. *niveum* (*Fon*), poses a serious threat to watermelon productivity. We previously characterized six antagonistic bacterial strains, including DHA6, capable of suppressing watermelon Fusarium wilt under greenhouse conditions. This study investigates the role of extracellular cyclic lipopeptides (CLPs) produced by strain DHA6 in Fusarium wilt suppression. Taxonomic analysis based on the *16S rRNA* gene sequence categorized strain DHA6 as *Bacillus amyloliquefaciens*. MALDI-TOF mass spectrometry identified five families of CLPs, i.e., iturin, surfactin, bacillomycin, syringfactin, and pumilacidin, in the culture filtrate of *B. amyloliquefaciens* DHA6. These CLPs exhibited significant antifungal activity against *Fon* by inducing oxidative stress and disrupting structural integrity, inhibiting mycelial growth and spore germination. Furthermore, pretreatment with CLPs promoted plant growth and suppressed watermelon Fusarium wilt by activating antioxidant enzymes (e.g., catalase, superoxide dismutase, and peroxidase) and triggering genes involved in salicylic acid and jasmonic acid/ethylene signaling in watermelon plants. These results highlight the critical roles of CLPs as determinants for *B. amyloliquefaciens* DHA6 in suppressing Fusarium wilt through direct antifungal activity and modulation of plant defense responses. This study provides a foundation for developing *B. amyloliquefaciens* DHA6-based biopesticides, serving as both antimicrobial agents and resistance inducers, to effectively control Fusarium wilt in watermelon and other crops.

## 1. Introduction

Beneficial microbes offer a promising and sustainable alternative to traditional chemical pesticides for the biological control of crop diseases [1,2]. Among these, bacteria from the genus *Bacillus* and *Pseudomonas* as well as fungi such as *Trichoderma* spp. have been extensively studied as biocontrol agents [1,2,3]. A multitude of microbial biocontrol agents has been developed using *Pseudomonas*, *Trichoderma*, and *Bacillus* species (spp.) and applied to manage fungal and bacterial diseases in crops [4]. *Bacillus* spp., due to their ubiquitous distribution in soil, rhizosphere, and plant surfaces, are the most exploited beneficial bacteria [1,5,6,7,8,9]. Specifically, *Bacillus subtilis*, *Bacillus velezensis*, and *Bacillus amyloliquefaciens* have demonstrated effective suppression of *Fusarium oxysporum* (*Fo*)-caused Fusarium wilt and root rot in a wide range of crop plants [9]. For instance, *B. subtilis* YB-04 provided remarkable efficacy (>90%) in controlling cucumber Fusarium wilt (*Fo* f. sp. *cucumerinum*) [10]. Similarly, *B. amyloliquefaciens* YN0904 and *B. subtillis* YN1419 conferred an efficacy of >80% in controlling banana Fusarium wilt (*Fo* f. sp. *cubense* tropical race 4) [11]. The application of *B. velezensis* F21 significantly suppressed watermelon Fusarium wilt (*Fo* f. sp. *niveum*; *Fon*) by 80.35% in the greenhouse and 65.81% in the field [12]. Moreover, seed treatment with *Bacillus tequilensis* PKDN31 and *Bacillus licheniformis* PKDL10 reduced (85%) the severity of tomato Fusarium wilt (*Fo* f. sp. *lycopersici*) [13]. These studies strongly support the use of beneficial *Bacillus* spp. as an effective strategy for managing Fusarium wilt in crops [9], a major threat to agriculture sustainability [14,15].

*Bacillus* spp., as biocontrol agents, can produce various bioactive metabolites that operate distinct mechanisms involved in direct antimicrobial activity, plant growth promotion, and defense response induction [16,17,18]. Among these, cyclic lipopeptides (CLPs), such as iturins, fengycins, bacillomycin, and surfactins, represent the most important class of bioactive compounds produced by *Bacillus* spp. [16,18]. For example, *B. amyloliquefaciens* DHA55 produces four CLPs, iturin A, fengycin, surfactin, and bacillomycin, while *B. velezensis* WB and Bs006 synthesize iturin A, fengycin, surfactin, and bacitracin [19,20,21]. These CLPs possess direct antimicrobial activity, making them potential candidates for controlling plant diseases. For example, iturin A from *B. amyloliquefaciens* significantly inhibited *Fon* and other pathogenic fungi [19,22], while fengycins produced by *B. amyloliquefaciens* PPL showed inhibitory effects against *Fo* f. sp. *lycopersici* [23]. CLPs derived from *B. velezensis* WB have been found to trigger oxidative stress and decrease toxin synthesis in *Fon*, thus conferring antifungal activity [20]. Moreover, volatile antifungal compounds such as phenylacetic acid and methylphenyl acetate produced by *B. mycoides* BM02, and myriocin produced by *B. amyloliquefaciens* LZN01, have been reported to inhibit the growth of *Fo* f. sp. *lycopersici* and *Fon*, respectively [24,25]. These facts suggest that *Bacillus* spp. can produce a wide range of antimicrobial compounds, including CLPs, which hold potential for development as biopesticides for direct application in the field for managing crop diseases [1,18].

Although the direct antifungal activity of CLPs is recognized as a primary mechanism in controlling plant diseases, they have also been demonstrated to stimulate an innate immune response in plants, called induced systemic resistance (ISR), against multiple biotic stresses [1,8]. For instance, plipastatin, surfactin, and mycosubtilin derived from *B. amyloliquefaciens* and *B. subtilis* have been shown to induce disease resistance in strawberry and grapevines against anthracnose (*Colletotrichum gloeosporioides*) and gray mold (*Botrytis cinerea*), respectively, *via* activating defense-related genes [26,27]. Similarly, CLPs produced by *B. amyloliquefaciens* subsp. *plantarum* were found to induce defense genes in lettuce against bottom rot fungus *Rhizoctonia solani* [28]. Further, *Bacillus*-produced phenylacetic acid activated ISR against Fusarium wilt in tomato and banana [29,30,31,32,33,34]. The CLPs-triggered ISR is generally dependent on the jasmonic acid (JA), ethylene (ET), or brassino-steroid signaling pathways that regulate a sophisticated network of defense-related genes in plants [29,31,35]. CLPs not only modulate defense-related genetic mechanisms but also boost the antioxidant defense capacity in plants. For example, a *B. velezensis* L-H15-produced CLP, P852, enhanced the basal immunity of faba beans against Fusarium wilt by activating antioxidant enzymes, such as catalase, superoxide dismutase, and peroxidase [36]. However, further investigations are required to elucidate the specific role of CLPs in inducing plant defense signaling pathways.

Watermelon Fusarium wilt, caused by the soil-borne root-infecting fungus *Fon*, is a devastating disease that can result in serious yield loss [37]. *Fon* can persist in the soil for extended periods, resulting in severe disease outbreaks, especially when mono-cropping the system. Therefore, it is crucial to implement effective strategies to manage Fusarium wilt and ensure the sustainable development of the watermelon industry. In a previous study, we characterized six antagonistic bacterial strains isolated from the rhizosphere of field-grown watermelon plants, which exhibited plant growth-promoting and Fusarium wilt-suppressing ability in watermelon [19]. In the present study, we aimed to elucidate the role and underlying mechanisms of *B. amyloliquefaciens* DHA6-derived CLPs in promoting plant growth and suppressing Fusarium wilt in watermelon. Our findings demonstrate that CLPs are critical determinants of the biocontrol capacity of *B. amyloliquefaciens* DHA6 against Fusarium wilt, which displayed not only direct antifungal activity against *Fon* but also performed the immunomodulatory role in watermelon plants. Thus, our study highlights the significance of CLPs as key bioactive compounds in the biocontrol of Fusarium wilt in watermelon by *B. amyloliquefaciens* DHA6, and provides a basis for developing more sustainable and eco-friendly strategies for crop protection.

## 2. Materials and Methods

### 2.1. Growth Conditions for Fon, DHA6, and Watermelon Plants

The *Fon* strain ZJ1 was cultured on potato dextrose agar (PDA; 200 g potato extract, 20 g glucose, 1 L ddH_2_O) [38], while the spore suspension (3 × 10^6^ spores/mL) used for inoculation was prepared by growing the fungus in mung bean broth (15 g mung bean extract, 1 L ddH_2_O) at 28 ± 2 °C and 150 rpm for 48 h, as previously described [38]. The bacterial strain DHA6 was maintained on a Luria-Bertani (LB; Sigma-Aldrich, St. Louis, MO, USA) plate. To extract CLPs, a single colony of DHA6 was inoculated into 100 mL LB broth in 250 mL Erlenmeyer flasks and incubated with shaking at 150 rpm and 28 ± 2 °C for 72 h.

Watermelon (*Citrullus lanatus* L. cv. Zaojia) seeds were surface sterilized by immersing in 3% sodium hypochlorite solution (Sigma-Aldrich, St. Louis, MO, USA) for 3 min, rinsed twice with ddH_2_O, and germinated under moist conditions at 26 °C. The germinated seeds were transplanted into pots (6 cm × 4 cm) filled with sterilized soil mixture (soil: organic manure: sand = 2:1:1, *w*/*w*). The plants were grown in a greenhouse under natural light at 32/18 °C (day/night) temperature and 70% humidity. At the two-true-leaf stage, plants were shifted into soil-filled pots (50 cm length × 25 cm width × 5 cm height) with 25 plants/pot.

### 2.2. Molecular Characterization of the Bacterial Strain DHA6

Genomic DNA was extracted from DHA6 cells using the MiniBEST Bacterial Genomic DNA Extraction Kit Ver3.0 (Takara, Dalian, China). The universal primer pair 27F (5′-AGAGTTTGATCATGGCTCAG-3′) and 1479R (5′-TACGGTTACCTTGTTACGACTT-3′) [39] was used to amplify a *16S rRNA* gene fragment, which was then commercially sequenced (Zhejiang Youkang Biotech, Hangzhou, China). The obtained sequences were trimmed, processed for contig formation, and the final sequence was aligned with its homologues using the ClustalX program [40]. A phylogenetic tree was constructed using the neighbor-joining method in the MEGA 11.0 software package [41]. 

### 2.3. Extraction, Purification, and Characterization of CLPs from Strain DHA6

The cell-free supernatant was collected from a 200 mL culture of strain DHA6, acidified using 2 M HCl (pH 2.0), and kept overnight at 4 °C. After incubation, CLPs were precipitated through centrifugation at 14,000 rpm for 20 min at 4 °C, re-dissolved in methanol (pH 7.0), and dried using a rotary vacuum [42]. The dried CLPs were subsequently dissolved in dimethyl sulfoxide (DMSO; Sigma-Aldrich, St. Louis, MO, USA) at 100 μg/mL for further experimental use.

The CLPs produced by strain DHA6 were characterized using matrix-assisted laser desorption ionization-time of flight (MALDI-TOF)-mass spectrometry (MS) method [43,44]. DHA6 was cultured in tryptic soy broth medium at 28 ± 2 °C for 48 h, and a single colony was suspended in a matrix solution (10 mg/mL cyano-4-hydroxycinnamic acid in 70% water, 30% acetonitrile, and 0.1% trifluoroacetic acid). The bacterial samples were homogenized, and 1 μL of the solution was spotted onto a MALDI-TOF MTP 384 objective plate (Bruker Daltonik GmbH, Leipzig, Germany). After air-drying, MALDI-TOF-mass spectra were recorded using a Bruker Ultraflextreme MALDI-TOF instrument (Bruker, Bremen, Germany) equipped with a Smartbeam laser operating at a 2-kHz repetition rate. Spectra were acquired in positive linear ion mode within the mass range of 300 to 3000 Dalton (Da).

### 2.4. Antifungal Activity of CLPs against Fon

The antifungal activity of CLPs against *Fon* mycelial growth was determined using the dual culture method [45,46]. PDA plates were prepared with CLPs added at final concentrations of 12.5, 25, 50, and 100 μg/mL, or with 100 μL of DMSO as control. *Fon* mycelial plugs (0.5 cm in diameter) were inoculated onto the plates, followed by incubation at 28 ± 2 °C for ~5 d. The inhibitory zones were measured to determine the extent of growth inhibition.

To evaluate the effect of CLPs on *Fon* spore germination, 1 mL *Fon* conidia suspension (10^3^ conidia/mL) was incubated with different concentrations of CLPs, 21, 24, 27, and 30 µg/mL, or with 100 μL of DMSO as control at 28 ± 2 °C for 24 h with shaking at 180 rpm. The spore germination rate was assessed by observing the appearance and growth of germ tubes.

### 2.5. Optical and Electronic Microscopy Observation

The viability of *Fon* mycelia and conidia was assessed using fluorescein diacetate (FDA; Yeasen Biotech, Shanghai, China) and propidium iodide (PI; Yeasen Biotech, Shanghai, China) staining methods [47]. Mycelia and conidia were treated with or without CLPs (30 µg/mL) for 12 h, collected by centrifugation at 1000 rpm for 10 min, and resuspended in 10 mM phosphate buffer saline (PBS, pH 7.4). After staining with FDA and PI at 25 °C for 15 min in the dark, fluorescent signals were observed under a Zeiss LSM 880 confocal laser microscope (Zeiss, Jena, Germany) at excitation/emission wavelengths of 488/500–550 nm for FDA and 561/600–650 nm for PI.

The impact of CLPs on *Fon* mycelial and conidial morphology was examined using scanning electron microscopy (SEM; Model TM-1000, Hitachi, Japan) and transmission electron microscopy (TEM; Model JEM–1230JEOL, Akishima, Japan) [48,49]. *Fon* mycelia and conidia were treated with or without CLPs (30 µg/mL) for 12 h and then collected by centrifugation at 1000 rpm for 10 min. The collected mycelia and conidia were pre-fixed overnight in 2.5% glutaraldehyde in 100 mM PBS (pH 7.0), post-fixed with 1% osmic acid for 1 h, and dehydrated using an ethanol gradient (30–100%). For SEM, the samples were incubated in an alcohol and isoamyl acetate (1:1, *v*/*v*) mixture for 30 min, treated in pure isoamyl acetate for 1 h, and dehydrated in a critical point dryer (Model HCP-2, Hitachi, Tokyo, Japan) with liquid CO_2_. After coating with gold-palladium, the samples were examined under SEM at 15 kV. For TEM, the dehydrated mycelia and conidia were embedded in epoxy resin, sectioned into ultrathin sections using an EM UC7 ultramicrotome (Leica Microsystems, Vienna, Austria), stained with uranyl acetate or lead citrate, and examined under TEM. Six samples from each treatment were used for TEM/SEM analysis, and at least 5 fields were examined for each sample.

### 2.6. Detection of Reactive Oxygen Species (ROS) Accumulation

ROS production was detected using the 2′,7′-dichlorodihydrofluorescein diacetate (DCFH-DA; Sigma-Aldrich, St. Louis, MO, USA) probe [50,51]. *Fon* mycelia and conidia were treated with or without CLPs (30 µg/mL) for 5 h, collected by centrifugation at 1000 rpm for 10 min, and resuspended in 10 mM PBS (pH 7.4). The samples were then treated with 10 mM DCFH-DA and incubated at 25 °C for 30 min in the dark. ROS levels were visualized using the Zeiss LSM 880 confocal laser microscope (Zeiss, Jena, Germany; excitation wavelength, 488 nm; emission wavelength, 535 nm).

### 2.7. Effect of CLPs on Plant Growth and Fusarium Wilt in Watermelon

Four treatments, including ddH_2_O (Mock), CLPs, *Fon*, and CLPs + *Fon*, were set up with three replicates in each treatment (25 plants/replicate). To avoid direct contact between CLPs and *Fon*, a plastic plate (50 cm length × 25 cm width × 5 cm height) system was used. Watermelon plants with two true leaves were transferred into a soil mixture in plastic pots and placed in a container tray for 10 d until the roots reached the tray across the holes on the bottom of the pots. The plants were root-treated with or without CLPs (10 µM) solution in ddH_2_O. After 24 h post-treatment, one set of the CLPs-treated and untreated plants was inoculated with *Fon* using the root-dipping method [52]. For this, 10 mL of *Fon* spore suspension (4 × 10^6^ spore/mL) or ddH_2_O (as a mock control) was applied to the plants by adding to the tank tray. The inoculated plants were covered with plastic membranes for 3 d to favor disease development [47]. Root samples were taken from three plants/treatment at 3 and 6 d after inoculation for the analysis of antioxidative enzyme activity and defense gene expression. The disease index was assessed using a 4-scale rating standard (0 = no symptoms, 1 = chlorosis, 2 = wilting, and 3 = death) [38] at 2 weeks post-inoculation. Additionally, growth parameters, including plant (aboveground part) height, root length, and fresh and dry weights of plants and roots, were measured as previously described [19].

### 2.8. Measurement of Antioxidative Enzyme Activity

Root samples were thoroughly washed with ddH_2_O and homogenized in liquid nitrogen. The homogenate (~100 mg) was then extracted in PBS (pH 7.8) containing 2.0 mM β-mercapto-ethanol (Sigma-Aldrich, St. Louis, MO, USA), 1.0 mM Ethylenediaminetetraacetic acid (EDTA; Sigma-Aldrich, St. Louis, MO, USA), and 1% (*w*/*v*) polyvinylpyrrolidone (Sigma-Aldrich, St. Louis, MO, USA). Following centrifugation at 12,000× *g* for 20 min at 4 °C, the resulting supernatant was collected as enzyme extract, and the activities of superoxide dismutase (SOD), peroxidase (POD), and catalase (CAT) were spectrophotometrically evaluated as described previously [53,54]. Briefly, CAT activity was measured in a reaction containing 2.9 mL 0.1% hydrogen peroxide (H_2_O_2_; Sigma-Aldrich, St. Louis, MO, USA) and 100 μL enzyme extract by recording the absorbance at 240 nm at 1 s intervals for 3 min. SOD reactions contained 1.5 mL 50 mM PBS (pH 7.8), 0.3 mL 130 mM methionine, 0.3 mL 1 mM EDTA-Na_2_, 0.3 mL 0.2 mM riboflavin, 0.3 mL 750 μM nitrogen blue tetrazolium, and 0.3 mL enzyme extract or 0.3 mL ddH_2_O as a blank. The reactions were exposed to 4000 lx of light for 10 min, and the absorbance at 560 nm was recorded. POD activity was determined in a reaction containing 1.0 mL 50 mM PBS (pH 7.0), 1.0 mL 0.3% H_2_O_2_, 0.9 mL 0.2% guaiacol, and 0.1 mL enzyme extract by recording the absorbance at 470 nm at 1 s intervals for 3 min.

### 2.9. RNA Extraction and RT-qPCR Analysis of Gene Expression

Total RNA was extracted from root samples using Trizol reagent (Vazyme Biotech, Nanjing, China) and then reverse transcribed into cDNA using HiScript super mix (Vazyme Biotech, Nanjing, China). For qPCR reactions, 10 μL 2 × AceQ qPCR SYBR Green Master Mix (Vazyme, Nanjing, China), 0.1 μg cDNA, and 7.5 pmol of each gene-specific primer were combined in a final volume of 20 μL. The qPCR reactions were run on a CFX96 real-time PCR detection system (Bio-Rad, Hercules, CA, USA) using the following reaction conditions: 94 °C for 5 min, followed by 45 cycles of 94 °C for 10 s, 55 °C for 20 s, and 72 °C for 30 s, and end at 72 °C for 5 min. Watermelon *ClGAPDH* was used as an internal control for data normalization [55], and the relative gene expression level was calculated using the 2^−∆∆Ct^ method. Gene-specific primers used for qPCR are listed in Table 1.

### 2.10. Experimental Design and Data Analysis

All experiments were conducted three times independently, with three replicates in each treatment. Statistical analysis was performed on the data obtained from three independent experiments using SPSS 14.0 software, and the difference among treatments was considered significant at *p*-value ≤ 0.05.

## 3. Results

### 3.1. Molecular Characterization of Strain DHA6

**Figure 1:**
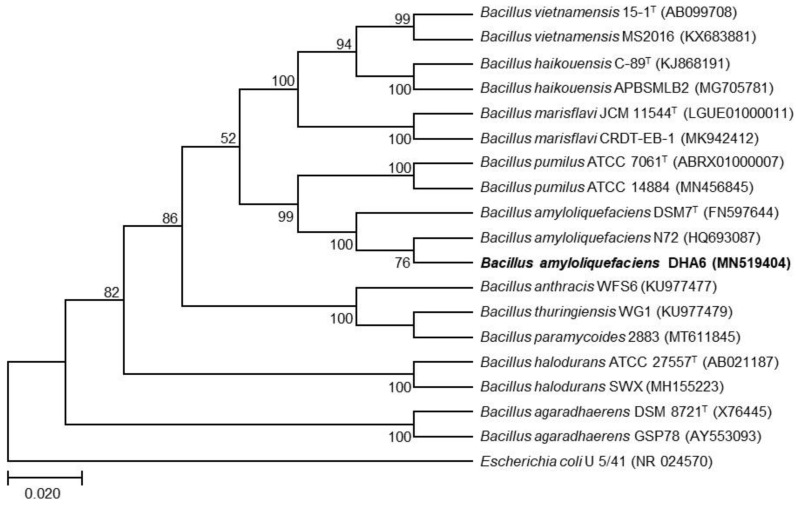
Molecular characterization of strain DHA6. The phylogenetic tree was constructed using the neighbor-joining method in MEGA11.0 software, comparing the *16S rRNA* gene sequence of strain DHA6 with those from other *Bacillus* species. The *16S rRNA* gene sequence of *Escherichia coli* U 5/41 (NR 024570) was used as an outgroup.

To characterize strain DHA6 taxonomically, a 1023 bp fragment of the *16S rRNA* gene was amplified and sequenced. Sequence alignment and phylogenetic analysis revealed a high similarity between the *16S rRNA* gene sequence of strain DHA6 (GenBank accession no. MN519404) and other *Bacillus* spp. The DHA6 *16S rRNA* sequence closely clustered with *B. amyloliquefaciens* N72, showing >99% sequence identity to strain N72 (Figure 1). Therefore, it is likely that strain DHA6 belongs to *B. amyloliquefaciens*.

### 3.2. Identification of CLPs Produced by B. amyloliquefaciens Strain DHA6

**Figure 2:**
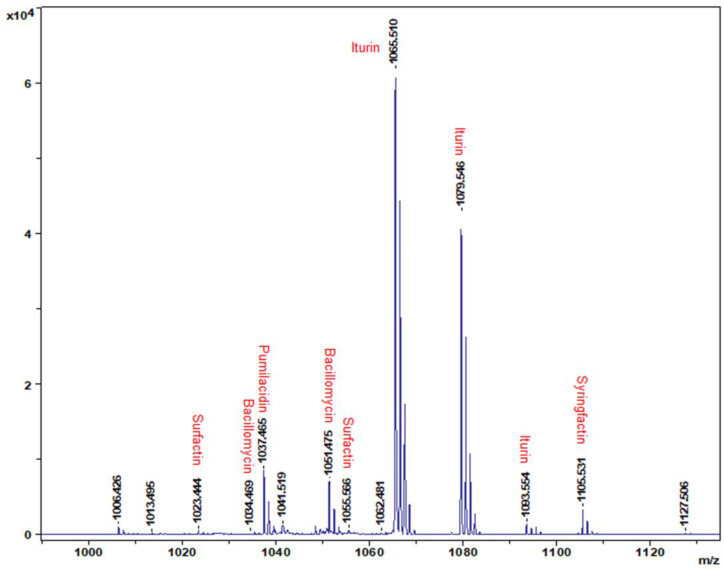
Identification of *B. amyloliquefaciens* DHA6-derived cyclic lipopeptides (CLPs) by the matrix-assisted laser desorption/ionization-time of flight approach. The spectrum displays the putative CLPs and their corresponding *m*/*z* values.

MALDI-TOF analysis identified different *B. amyloliquefaciens* DHA6-produced CLPs, with standard peaks ranging from 1000 to 1120 *m*/*z* (Figure 2). The highest peak in the MALDI-TOF profile typically corresponds to non-ribosomal CLPs. *B. amyloliquefaciens* DHA6 was found to produce five classes of CLPs, namely iturin (*m*/*z*:1065.510, 1079.546, and 1093.554), surfactin (*m*/*z*:1055.566, and 1023.444), bacillomycin (*m*/*z*:1034.469, and 1051.475), pumilacidin (*m*/*z*:1037.465), and syringfactin (*m*/*z*:1105.531) (Figure 2). These data indicate that *B. amyloliquefaciens* DHA6 can produce diverse CLPs, which could contribute to its antifungal activity against different phytopathogenic fungi [19].

### 3.3. Plant Growth Promotion and Fusarium Wilt Suppression in Watermelon by CLPs

**Figure 3:**
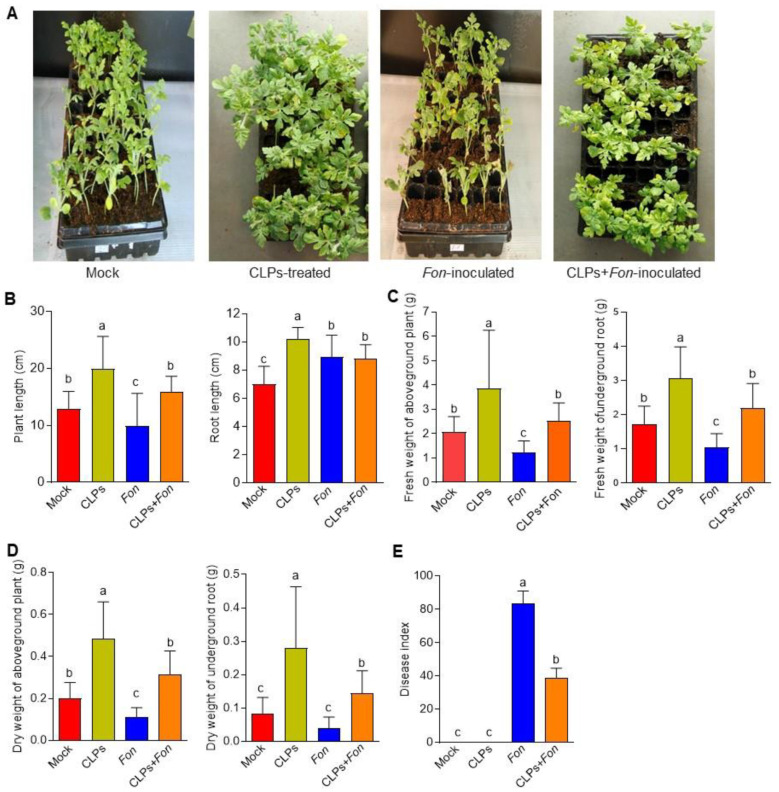
Plant growth promotion and Fusarium wilt suppression by *B. amyloliquefaciens* DHA6-produced cyclic lipopeptides (CLPs). (**A**) The growth and disease phenotype of CLPs (30 μg/mL)-treated and untreated watermelon plants after infection with or without *F. oxysporum* f. sp. *niveum* (*Fon*). (**B**,**C**) Plant height and root length; (**C**,**D**) Fresh and dry weights of aboveground plants and roots. (**E**) Disease index. The experiments in (**A**) were independently performed three times, and similar results were obtained. Data presented in (**B**–**E**) are the means ± standard deviation from three independent experiments, and different letters indicate significant differences among the treatments inferred using one-way analysis of variance (*p*-value ≤ 0.05).

We previously demonstrated that *B. amyloliquefaciens* DHA6 could improve plant growth and suppress watermelon Fusarium wilt [19]. In this study, we investigated the potential roles of *B. amyloliquefaciens* DHA6-produced CLPs in promoting plant growth and suppressing Fusarium wilt in watermelon. Repeated greenhouse experiments showed a significant improvement in the growth performance of uninfected CLPs-treated plants compared to untreated healthy controls (Figure 3A), which was evident from the increased plant height (35.17%), root length (31.37%), as well as the fresh and dry weight of aboveground plants (46.29% and 58.88%, respectively) and roots (43.41% and 69.82%, respectively) (Figure 3B–D).

In disease assays, *Fon*-inoculated plants showed a higher disease index (83.33) and typical Fusarium wilt disease symptoms, including yellowing and wilting of leaves, while the *Fon*-inoculated CLPs-pretreated plants displayed significantly less disease severity index (38.67) and disease symptoms, such as slight chlorosis (Figure 3A), leading to a reduction of 54.6% in disease index (Figure 3E). During the experiments, no obvious disease symptom was observed in mock- and CLPs-pretreated plants (Figure 3E). Furthermore, the growth parameters of the *Fon*-infected plants were dramatically reduced compared to the uninfected control plants (Figure 3B–D). In contrast, the growth of *Fon*-inoculated CLPs-pretreated plants was comparable to the control healthy plants (Figure 3B–D). These results indicate that *B. amyloliquefaciens* DHA6-produced CLPs can promote plant growth and suppress Fusarium wilt in watermelon under greenhouse conditions.

### 3.4. Antifungal Activity of CLPs against Fon

**Figure 4:**
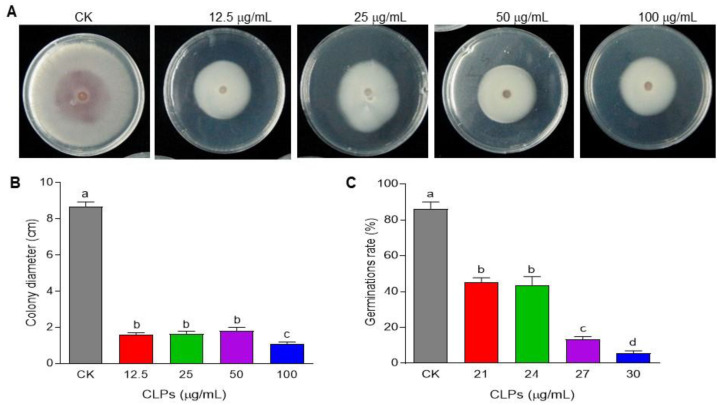
Inhibition of *F. oxysporum* f. sp. *niveum* (*Fon*) mycelial growth and spore germination by *B. amyloliquefaciens* DHA6-produced cyclic lipopeptides (CLPs). (**A**,**B**) Phenotype (**A**) and colony size (**B**) of *Fon* grown in the presence of different CLPs concentrations. (**C**) Inhibition of spore germination of *Fon* by CLPs. The experiments in (**A**) were independently performed three times, and similar results were obtained. Data presented in (**B**,**C**) are the means ± standard deviation from three independent experiments, and different letters indicate a significant difference among the treatments inferred using one-way analysis of variance (*p*-value ≤ 0.05).

To explore the mechanisms underlying *B. amyloliquefaciens* DHA6-mediated suppression of watermelon Fusarium wilt, as previously reported [19], we examined the impact of CLPs on mycelial growth and spore germination in *Fon*. In these experiments, the specific CLPs concentrations for mycelial growth and spore germination were selected based on pilot experiments that indicated varying sensitivities of *Fon* mycelia and conidia to CLPs. Overall, CLPs significantly inhibited the mycelial growth of *Fon* (Figure 4A), resulting in smaller mycelial colonies of *Fon* on CLPs-supplemented PDA (Figure 4B). CLPs inhibited the mycelial growth of *Fon* in a dosage-dependent manner, with a maximum antifungal activity observed at 100 μg/mL CLPs, leading to an 87.3% inhibition of *Fon* mycelial growth (Figure 4A,B). Furthermore, CLPs also inhibited the germination of *Fon* spores in a dosage-dependent manner. At 30 µg/mL, CLPs inhibited the spore germination, resulting in a 93.8% reduction compared to untreated spores (Figure 4C). These results indicate that *B. amyloliquefaciens* DHA6-produced CLPs exhibit remarkable antifungal activity against *Fon*, with the effectiveness varying based on the dosage, and contribute to the biocontrol potential of *B. amyloliquefaciens* DHA6 in suppressing watermelon Fusarium wilt.

### 3.5. Inhibitory Effect of CLPs on Fon Viability

**Figure 5:**
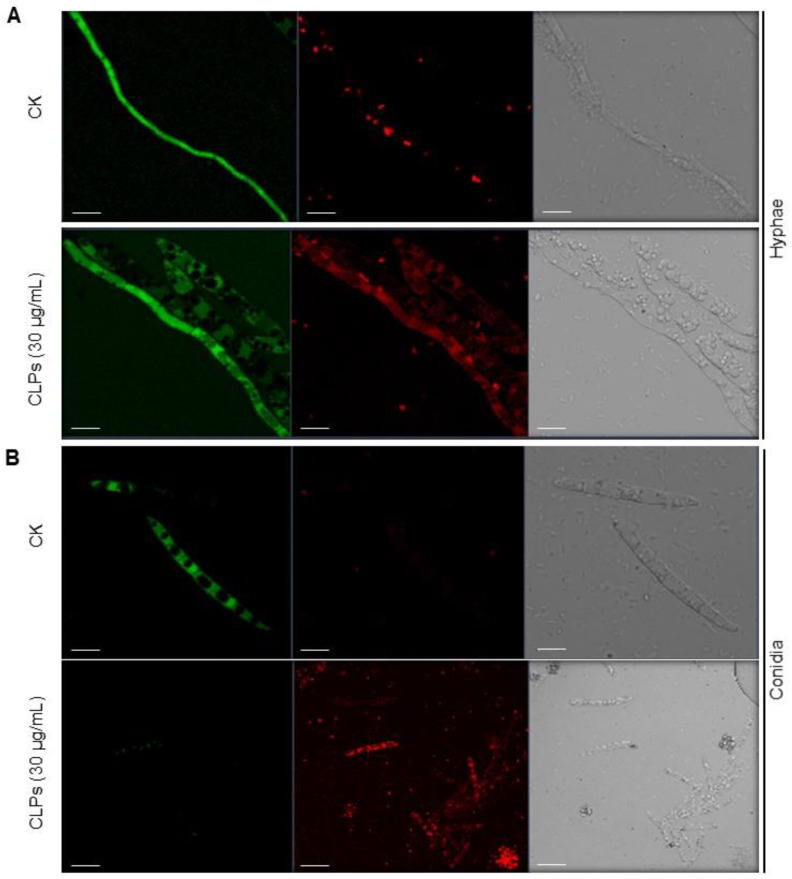
*B. amyloliquefaciens* DHA6-produced cyclic lipopeptides (CLPs) affect *F. oxysporum* f. sp. *niveum* (*Fon*) viability. *Fon* mycelia (**A**) and conidia (**B**) were treated with or without CLPs (30 μg/mL) and stained with fluorescein diacetate (FDA) and propidium iodide (PI) 12 h after treatment. Green and red fluorescent signals were detected using a confocal laser microscope, with excitation at 488 nm for FDA and 561 nm for PI, respectively. Scale bar, 10 µm. The experiments were independently performed three times with similar results, and data from one representative experiment are shown.

To explore the putative causes of the antifungal activity of CLPs, we examined their effect on the viability and ultrastructural changes of *Fon* through FDA and PI staining. FDA generates green fluorescence after entering living cells, serving as an indicator of living cells, while PI is a nucleus-specific dye for detecting apoptosis, serving as a marker of dead cells [50,56]. Without CLPs treatment, *Fon* mycelia and conidia showed strong FDA-generated green fluorescence, indicating normal morphology and intact structures, with minimal red fluorescence in mycelia only, representing dead or damaged fungal cells (Figure 5A,B). However, the CLPs-treated *Fon* mycelia and conidia exhibited bright red fluorescence, indicating disrupted morphology and structure. After staining, the CLPs-treated mycelia showed diffuse red and green fluorescence, but the CLPs-treated conidia no longer exhibited green fluorescence (Figure 5A). These observations suggest that *B. amyloliquefaciens* DHA6-produced CLPs affect the integrity of *Fon* cells, leading to cell damage and reduced viability.

**Figure 6:**
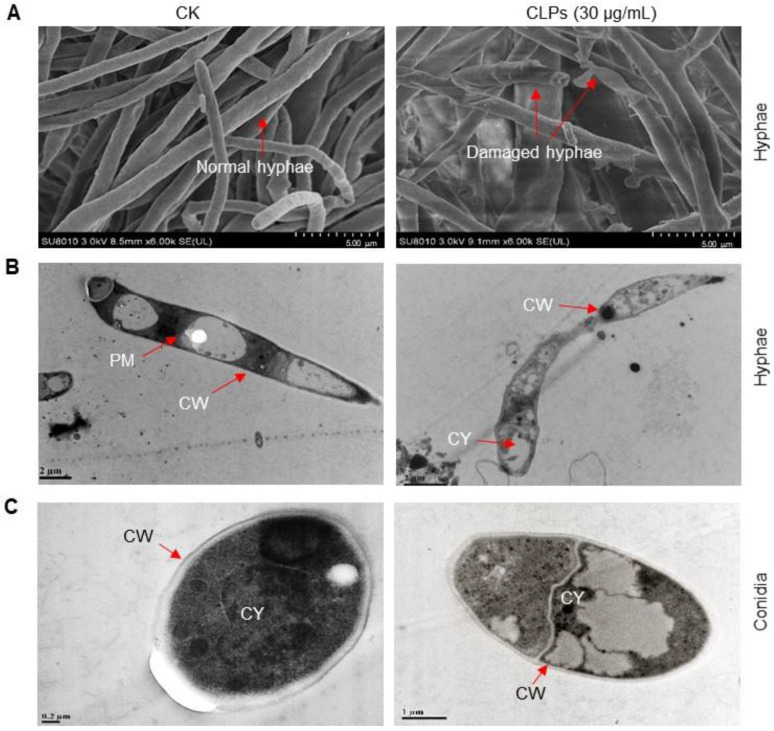
Ultrastructural changes in *F. oxysporum* f. sp. *niveum* (*Fon*) mycelia and conidia caused by *B. amyloliquefaciens* DHA6-produced cyclic lipopeptides (CLPs). (**A**) Scanning electron microscopic images showing the impact of CLPs (30 μg/mL) on the mycelial morphology of *Fon*. (**B**,**C**) Transmission electron microscopic images showing the ultrastructural changes in the mycelia (**B**) and conidia (**C**) with or without CLPs (30 µg/mL) treatment. The ultrastructural changes in the mycelia and conidia of *Fon* were examined at 12 h after CLPs treatment. CW, cell wall; CY, cytoplasm; PM, plasma membrane. Scale bars are shown at the bottom right for SEM images and the bottom left for TEM images. The experiments were independently performed three times with similar results, and data from one representative experiment are shown. Red arrows represent normal and damaged *Fon* hyphal and cellular structures.

To further investigate the effect of CLPs on the morphology and integrity of *Fon*, we examined ultrastructural changes in mycelia and conidia through SEM and TEM. SEM images revealed that untreated mycelia had regular, intact and column-like trunks, while the CLPs-treated mycelia showed obvious structural damages, such as disintegrated and collapsed mycelial structures (Figure 6A). Similarly, TEM images showed that untreated mycelia and conidia maintained normal cellular morphology and structure, with intact cell walls, plasma membranes, and cytoplasm (Figure 6B,C). In contrast, CLPs-treated mycelia and conidia displayed abnormal morphology and structure, with damaged plasma membrane and cell wall (Figure 6B,C). These results indicate that *B. amyloliquefaciens* DHA6-produced CLPs can disrupt the cellular structures and compromise the integrity of *Fon*, thereby influencing its viability.

### 3.6. Induction of ROS Accumulation in Fon by CLPs

**Figure 7:**
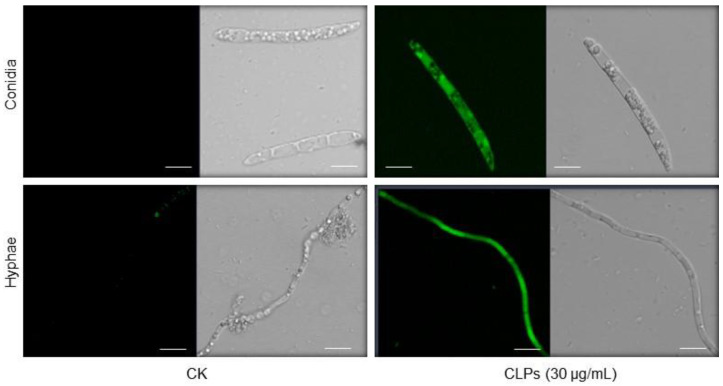
Reactive oxygen species (ROS) accumulation in *F. oxysporum* f. sp. *niveum* (*Fon*) mycelia and conidia triggered by *B. amyloliquefaciens* DHA6-produced cyclic lipopeptides (CLPs). The *Fon* mycelia and conidia were treated with CLPs (30 µg/mL) or dimethyl sulfoxide (control), and ROS accumulation was detected by 2′,7′-dichlorodihydrofluorescein diacetate staining. Scale bar, 10 µm. The experiments were separately performed three times with similar results, and data from one representative experiment are shown.

To gain insights into the physiological and biochemical mechanisms underlying CLPs-induced structural damages and antifungal activity against *Fon*, we assessed the effect of CLPs on ROS accumulation in the fungus using the DCFH-DA staining method. Results indicated that untreated *Fon* mycelia and conidia showed no significant green fluorescence, while CLPs-treated mycelia and conidia exhibited strong green fluorescence, reflecting higher ROS accumulation (Figure 7). These data indicate that *B. amyloliquefaciens* DHA6-produced CLPs can induce ROS accumulation in *Fon*, resulting in structural disintegration.

### 3.7. Enhancement of Antioxidative Capacity and Upregulation of Defense Gene Expression by CLPs

**Figure 8:**
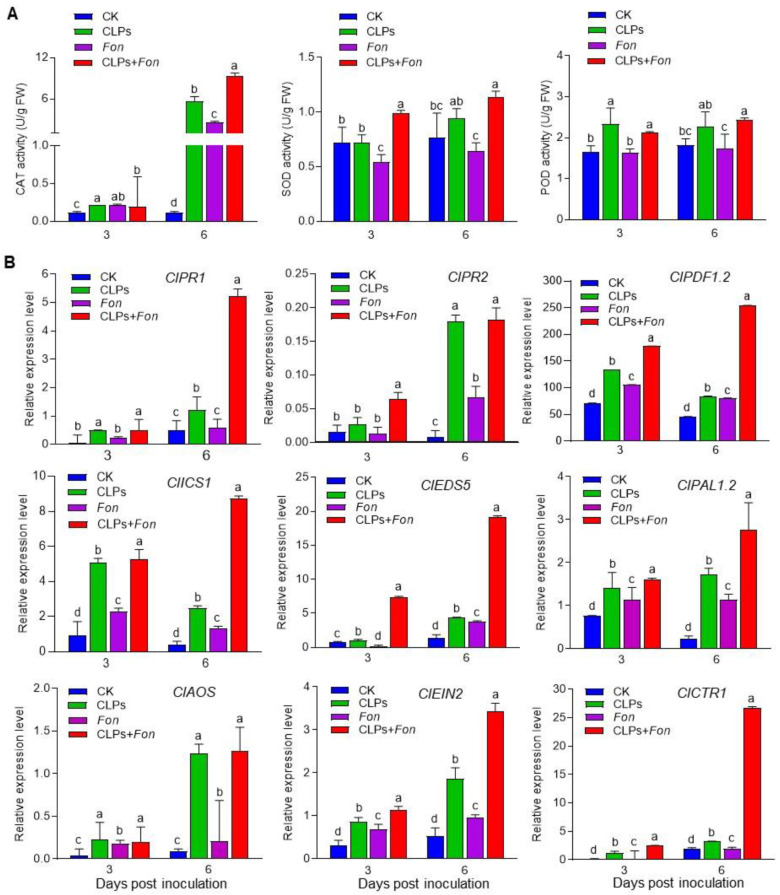
Effect of *B. amyloliquefaciens* DHA6-produced cyclic lipopeptides (CLPs) on antioxidative enzyme activities and expression of defense genes in watermelon plants. (**A**) Activities of catalase (CAT), superoxide dismutase (SOD), and peroxidase (POD) in *F. oxysporum* f. sp. *niveum* (*Fon*)-infected and uninfected watermelon plants pretreated with or without CLPs (30 μg/mL). (**B**) Expression levels of selected defense-related genes in *Fon*-infected and uninfected watermelon plants pretreated with or without CLPs (30 μg/mL). Data presented are the means ± standard deviation from three independent experiments, and different letters indicate a significant difference among the treatments inferred using one-way analysis of variance (*p*-value ≤ 0.05).

We further investigated the effect of *B. amyloliquefaciens* DHA6-produced CLPs on the defense response in watermelon plants to decipher the Fusarium wilt-suppressing mechanism. We examined the activities of CAT, SOD, and POD, three main ROS scavenging enzymes, in CLPs-pretreated plants before and after *Fon* infection. The activities of these antioxidant enzymes were significantly induced in CLPs-pretreated uninfected plants compared to untreated healthy plants at 3 dpi (except SOD), while CLPs-pretreated healthy plants showed significantly higher CAT level (97.98%) than untreated healthy controls at 6 dpi (Figure 8A). In *Fon*-infected plants, the SOD activity was significantly decreased by 24.65% at 3 dpi, while CAT activity was markedly increased by 95.62% at 6 dpi compared to untreated healthy plants (Figure 8A). In CLPs-pretreated *Fon*-infected plants, the activities of these enzymatic antioxidants were significantly higher compared to untreated *Fon*-infected plants at 3 dpi (except CAT) and 6 dpi (Figure 8A). These results indicate that *B. amyloliquefaciens* DHA6-produced CLPs can strengthen the antioxidative capacity by boosting CAT and SOD activities in watermelon plants, particularly in response to *Fon* infection.

To understand the molecular mechanism underlying the suppression of Fusarium wilt by *B. amyloliquefaciens* DHA6-produced CLPs, we analyzed the expression changes of selected defense-related genes in watermelon plants following CLPs treatment and *Fon* infection. In healthy plants, the expression levels of defense genes (*ClPR1*, *ClPR2*, and *ClPDF1.2*), salicylic acid (SA) signaling genes (*ClICS1*, *ClEDS5*, and *ClPAL2*), and JA/ET signaling genes (*ClAOS*, *ClEIN2*, and *ClCTR1*) were significantly upregulated in CLPs-pretreated healthy plants compared to untreated healthy controls (Figure 8B). In diseased plants, *Fon* infection induced the expression of these genes compared to untreated healthy plants, but their levels (except *ClEDS5*, *ClPAL1.2*, and *ClEIN2* at 6 d) were lower than those in CLPs-treated healthy plants (Figure 8B). In CLPs-pretreated infected plants, the expression levels of these genes were significantly upregulated compared to untreated/CLPs-pretreated healthy and untreated *Fon*-infected plants (Figure 8B). These results suggest that *B. amyloliquefaciens* DHA6-produced CLPs can induce the expression of genes involved in both SA and JA/ET signaling and defense response, especially upon *Fon* infection.

## 4. Discussion

*Bacillus* spp. employ multiple mechanisms to suppress phytopathogens and improve plant growth [1,2]. These mechanisms include direct and indirect antagonistic approaches, such as competition for nutrients and space, production of antimicrobial agents, and induction of plant defense networks. Among these, the production of antimicrobial metabolites is the primary inhibitory mechanism of *Bacillus* spp. [1,5]. In a previous study, we characterized six antagonistic watermelon rhizosphere colonizing bacterial strains, DHA4, DHA6, DHA10, DHA12, DHA41, and DHA55, which displayed substantial potential in promoting plant growth and suppressing watermelon Fusarium wilt [19]. In the present study, we investigated the underlying plant growth-promoting and disease-suppressing mechanism of strain DHA6, which previously provided significant protection against Fusarium wilt [19]. Molecular and biochemical characterization revealed that strain DHA6, belonging to *B. amyloliquefaciens*, produces antifungal CLPs (Figure 1). Our results showed that *B. amyloliquefaciens* DHA6-produced CLPs suppressed watermelon Fusarium wilt by inhibiting its hyphal growth and spore germination. Moreover, CLPs purified from DHA6 strain induced significant structural damage and ROS accumulation in *Fon*, reducing viability. These findings suggest that *B. amyloliquefaciens* DHA6-produced CLPs can be an effective alternative to conventional fungicides for managing *Fon*-induced wilt disease.

*B. amyloliquefaciens* strains have previously demonstrated biocontrol potential against Fusarium wilt in cucumber, tomato, and banana [11,57,58]. The production of bioactive compounds, including CLPs, is recognized as a crucial factor contributing to their biocontrol potential against crop diseases [16,17,18]. *Bacillus* spp. have been found to produce different CLPs, such as iturin, iturinA, bacillomycin D, and fengycin, which are well known for their disease-controlling ability [16,17,18,19]. In the present study, MALDI-TOF MS analysis revealed that *B. amyloliquefaciens* DHA6 produces five families of CLPs, i.e., iturin, iturinA, bacillomycin D, pumilacidin, and fengycin (Figure 2), consistent with CLPs profiles observed in other *B. amyloliquefaciens* strains, such as DHA55, NCPSJ7, YN201732, and PPL [19,22,23,59]. Pot experiments revealed that CLPs, similar to their source *B. amyloliquefaciens* DHA6 [19], possess significant potential in promoting plant growth and suppressing Fusarium wilt in watermelon (Figure 3), indicating that the production of CLPs is a prominent mechanism of *B. amyloliquefaciens* DHA6 involved in plant growth promotion and biocontrol of Fusarium wilt. Similarly, previous studies have demonstrated that iturin A- and surfactin A-enriched CLPs derived from *B. subtilis* BS-1 and *B. amyloliquefaciens* S76-3 have been shown to suppress kiwifruit rot (*Botryosphaeria dothidea*), rice bakanae disease (*Fusarium moniliforme*), wheat head scab (*Fusarium graminearum*), and lettuce Fusarium wilt [47,60,61,62]. Thus, antagonistic bacteria employ different antimicrobial CLPs as a primary mechanism for the biocontrol of crop diseases [16].

Previously, *B. amyloliquefaciens* DHA6 displayed antifungal activity against five different fungi, including *Fon*, *Didymella bryoniae* (causing gummy stem blight on cucurbits), *Sclerotinia sclerotiorum* (causing stem rot on a wide range of plants), *F. graminearum*, and *R. solani* (causing damping-off in a wide range of plants) [19]. This study found that CLPs from *B. amyloliquefaciens* DHA6 significantly inhibited mycelial growth and conidia germination of *Fon* (Figure 4), providing mechanistic insights into the direct antifungal activity of *B. amyloliquefaciens* DHA6 against phytopathogenic fungi. Previous studies have reported that CLPs purified from different *B. amyloliquefaciens* strains, such as fengycins and iturins, inhibited mycelial growth and spore germination of *Fo* in vitro [21,22,23,63,64]. Iturins produced by *Bacillus* spp. have been well known for their strong antifungal activity against *Fo* [63,64]. *B. amyloliquefaciens* DHA6 produced at least three types of iturins (Figure 2), which caused damage to the mycelia and conidia, disrupting the plasma membrane and cell wall, affecting *Fon* viability (Figure 4, Figure 5 and Figure 6). Similarly, CLPs produced by *B. velezensis* WB caused morphological changes, such as surface subsidence and cytoplasmic shrinkage, in *Fon* [20], while bacillomycin D, produced by *B. amyloliquefaciens* FZB42, induced disruptions in the plasma membrane and cell wall, leading to cell death of mycelia and conidia in *F. graminearum* [50]. The *B. amyloliquefaciens* DHA6-produced CLPs triggered a significant accumulation of ROS in the mycelia and conidia of *Fon* (Figure 7). These results align with previous observations indicating that iturins, fengycins, bacillomycin D and other CLPs triggered excessive ROS accumulation in *Fon*, *F. graminearum*, *Verticillium dahliae*, *Magnaporthe oryzae*, and *S. sclerotiorum* [20,50,65,66,67]. Although a physiological level of ROS is critical as an intracellular messenger in many biological events, the excessive accumulation of ROS can cause oxidative stress and severe cellular damage by disrupting cellular integrity. Thus, the disruption of ROS homeostasis is one of the physiological and biochemical mechanisms through which CLPs induce cell death and exhibit antifungal activity against different phytopathogenic fungi.

In addition to the direct antimicrobial activity, certain *Bacillus* spp., including *B. subtilis* and *B. amyloliquefaciens*, have been shown to strengthen plant defenses by priming ISR [7,68]. Bioactive compounds produced by different *Bacillus* spp., including CLPs, have been identified as key elicitors of ISR [7,69]. For example, iturins, surfactins, and fengycins produced by different *Bacillus* spp. have been found to induce significant ISR-mediated protection in various crop plants against different fungal infections [27,70,71,72]. In the present study, pretreatment with the *B. amyloliquefaciens* DHA6-produced CLPs, containing iturins, surfactins, pumilacidin, and fengycins (Figure 2), significantly reduced Fusarium wilt in watermelon (Figure 3). This reduction can be attributed to an enhanced defense response primed by CLPs in the plants, as direct contact between CLPs and *Fon* was avoided using a specific approach. The induction of plant defense responses by CLPs was further confirmed by the increased activity of antioxidative enzymes, i.e., CAT, SOD, and POD (Figure 8A), in watermelon plants upon CLPs pretreatment, supporting the notion that CLPs pretreatment induced plant innate defense mechanisms to suppress Fusarium wilt development. Similar results have been reported in infected watermelon and tomato plants, where *B. velezensis* F21 and *B. amyloliquefaciens* Oj-2.16 induced the activities of CAT, POD, and SOD through the production of antioxidant-triggering CLPs [12,73]. The plant immune system is believed to rely on coordinated signaling pathways, including SA, JA, and ET pathways, to mount an effective defense response against pathogen attack [12]. *Bacillus*-primed ISR has been shown to involve the JA/ET and SA signaling pathways and multiple early signaling events, such as MAPK cascades and ROS [26,28,74,75,76,77]. In lettuce, *B. amyloliquefaciens* FZB42-produced surfactin, and other immunomodulatory CLPs triggered immunity against *R. solani* by inducing the expression of defense-related genes, including *LsPDF1.2*, *LsPR1,* and *LsLOX* [28], while fusaricidin, purified from *Paenibacillus polymyxa* WLY78, induced SA-specific ISR to suppress Fusarium wilt in cucumber [78]. Consistent with these findings, we observed significant upregulation of SA-signaling/responsive, JA/ET signaling, and JA-responsive genes by CLPs, with or without *Fon* infection (Figure 8B), indicating their potential to activate a broad-spectrum defense response in watermelon plants. Therefore, it is likely that *B. amyloliquefaciens* DHA6-produced CLPs activate defense responses through the SA and JA/ET signaling pathways, thereby protecting watermelon plants from *Fon* infection.

## 5. Conclusions

In this study, the antagonistic bacterium *B. amyloliquefaciens* DHA6 was found to produce five families of antifungal CLPs, i.e., iturin, surfactin, bacillomycin, syrignfactin, and pumilacidin, which showed inhibitory effects against *Fon*. These CLPs inhibited mycelial growth and spore germination of *Fon* by promoting ROS accumulation and disrupting structural integrity of the pathogen. Furthermore, under greenhouse conditions, *B. amyloliquefaciens* DHA56-produced CLPs displayed plant growth-promoting activity and effectively suppressed Fusarium wilt in watermelon. This was accompanied by the activation of defense responses, as evidenced by enhanced activity of antioxidative enzymes and upregulation of genes involved in SA and JA/ET signaling pathways in watermelon plants. Our findings suggest that CLPs play a crucial role in the biocontrol capacity of *B. amyloliquefaciens* DHA6 against Fusarium wilt, acting through their direct antifungal activity and ability to induce plant defense responses. The potential of *B. amyloliquefaciens* DHA6-produced CLPs in promoting plant growth and suppressing Fusarium wilt in watermelon provides a basis for the development of *B. amyloliquefaciens* DHA6-based biopesticides, which can serve as both antimicrobial agents and resistance inducers, offering sustainable protection to important crops against disease damage under field conditions.

## Figures and Tables

**Table 1:** Gene-specific primers used in this study.

**Primers**	**Sequences (5′–3′)**
ClPR1-rt-1F	CTTGAGCTTTGCCATGCTGC
ClPR1-rt-1R	GCGTTGGTTGGCATATTGTCG
ClPR2-rt-1F	AACAACCTTCCAACCCAAAGAG
ClPR2-rt-1R	ATTCTTTGGAGGTCGGTATTGG
ClICS1-rt-1F	TAGGCAAAATTCAGCCACCG
ClICS1-rt-1R	AAGCTACGGCTGCTGGAATG
ClEDS5-rt-1F	GTGGCTTCACTTCATCTTCGTTC
ClEDS5-rt-1R	GCTGAGAAGTGAAGGAAGCGAG
ClAOS-rt-1F	TTCAACCCCACTTGCGATTTC
ClAOS-rt-1R	GATTGGGCGTAGGGAAACG
ClPDF1.2-rt-1F	GCTGCAATTTTGTTGCTCCTC
ClPDF1.2-rt-1R	TGTCCTTGCGTCTGTCACCA
ClCTR1-rt-1F	CCATTGTTGGCTTCCCTTATTG
ClCTR1-rt-1R	CGATGCTTGTGAAGGATGGG
ClEIN2-rt-1F	CTGCATACAACTCATCAGTCGGG
ClEIN2-rt-1R	CCACTTTCCAGGGTCAACATAAC
ClPAL2-rt-1F	TTGCGCCATTACTACTCATCCTG
ClPAL2-rt-1R	CGACCATGCGCTTCACCTC
qClGAPDH-F	ATGGGCAAAGTTAAGATCGGCATCA
qClGAPDH-R	CCAATTCGATATCATCACTCTGC

## Data Availability

All the data are present inside manuscript file.
